# Additive effects of intensive BP control and ACE inhibition on suppression of proteinuria in patients with CKD

**DOI:** 10.1038/s41371-023-00823-z

**Published:** 2023-03-25

**Authors:** Rafael Lopez, Timothy Copeland, Charles McCulloch, Elaine Ku

**Affiliations:** 1 School of Medicine, University of California San Francisco, San Francisco, CA USA; 2 Division of Nephrology, Department of Medicine, University of California San Francisco, San Francisco, CA USA; 3 Department of Epidemiology and Biostatistics, University of California San Francisco, San Francisco, CA USA

**Keywords:** Prognosis, Chronic kidney disease

## Abstract

The synergistic effects of RAAS inhibition and intensive blood pressure lowering in reducing proteinuria have not been well studied. We aimed to study this effect using data from the AASK Trial where our data suggest there is an additive and synergistic effect between RAAS inhibition and intensive blood pressure inhibition in reducing proteinuria.

## To the Editor:

Previous studies have shown that renin-angiotensin-aldosterone system (RAAS) inhibitors and intensive blood pressure (BP) lowering are both effective in lowering proteinuria [1–4]. However, up titration of RAAS inhibitors can be limited due to drug-related side effects such as hyperkalemia, and residual proteinuria can be present even with maximal doses of RAAS inhibitors [5]. Intensive BP lowering may also be effective in reducing proteinuria, but the comparative effects of RAAS blockade and intensive BP lowering used as either independent versus synergistic interventions to lower proteinuria have not been well characterized [3, 6]. Our objective was to quantify the effect of RAAS inhibition, with or without intensive BP lowering on the reduction of proteinuria leveraging trial data from the African American Study of Kidney Disease and Hypertension (AASK) Trial.

The data in this study were derived from the AASK Trial which are publicly available in the NIDDK Central Repository. Briefly, the AASK Trial was a 2 × 3 factorial design trial which tested the effect of two BP targets (mean arterial pressure 102–107 mmHg versus <92 mmHg) and three different BP agents (an angiotensin-converting enzyme inhibitor (ACE-I, ramipril), a calcium channel blocker (CCB) (amlodipine), and a beta-blocker (metoprolol)) on the progression of kidney disease. To achieve the assigned BP targets, AASK Trial permitted the use of other anti-hypertensive agents besides the randomized intervention [7, 8]. Our primary analysis only included patients with baseline proteinuria (defined by AASK as having a protein/creatinine ratio (PCR) > 0.22 g/g [9].

Our primary exposures were modeled as a six-category variable that accounted for both the randomly assigned BP target and agent, treating those receiving ACE-I and usual BP control as the reference group. The primary outcome in our study was the ratio of proportional change in PCR between baseline and month 6 compared to the reference group. The rationale for examining change in proteinuria over a 6-month period was because the AASK Trial aimed to achieve the assigned BP target over this timeframe and re-ascertained proteinuria at month 6 of study [7].

Our primary analysis was unadjusted given the preservation of randomization in the definition of our exposure. However, in secondary analysis, we also included a minimally adjusted model (Model 1) adjusting only for baseline proteinuria, and a fully adjusted model (Model 2) that was additionally adjusted for baseline age, sex, estimated glomerular filtration rate, and systolic (BP).

Although 358 patients were eligible for inclusion, 109 patients did not have PCR measured at 6 months post-randomization and were excluded; 2 developed end stage kidney disease and 7 died in the interim. For the 240 participants included in our analysis, mean age was 51 years, 62.9% were men, mean estimated glomerular filtration rate was 29 ml/min/1.73 m^2^, mean systolic blood pressure (BP) was 154 mmHg, and mean PCR was 0.65 g/g. These baseline characteristics were similar across all six groups.

The proportional change in PCR between baseline and month 6 in each of the six groups is shown in Table 1. Those randomly assigned to usual BP control and beta-blockade did not have a statistically significant difference in the proportional change in proteinuria (29% higher [95% CI 0.84, 1.99]) compared to the reference category. In contrast, the usual BP control and CCB group had a 168% increase in proteinuria [95% CI 1.57, 4.59] compared to the reference group (Table 1). Findings were similar in adjusted Models 1 and 2 (Table 1).

**Table 1:** Change in logarithm of proteinuria between baseline and month 6 by intensity of BP control and BP agent in the AASK Trial.

		PCR at Baseline (g/g) and IQR	PCR at 6 months (g/g) and IQR	Unadjusted ratio of proportional change in PCR compared with the reference (Usual BP + ACE-I)		Model 1^a^		Model 2^b^	
	*N*			Ratio	*p* value	Ratio	*p* value	Ratio	*p* value
Randomized assignment									
Usual BP arm ACE-I^c^	47	0.82 [0.37, 1.37]	0.52 [0.16, 1.33]	Reference		Reference		Reference	
Usual BP arm CCB^d^	22	0.71 [0.41, 1.42]	1.23 [0.65, 2.43]	2.68 [1.57, 4.59]	<0.001	2.68 [1.57, 4.59]	<0.001	2.59 [1.55, 4.35]	<0.001
Usual BP arm Beta-Blocker^e^	46	0.53 [0.38, 1.18]	0.50 [0.20, 1.31]	1.29 [0.84, 1.99]	0.24	1.26 [0.82, 1.95]	0.29	1.33 [0.88, 2.01]	0.18
Intensive BP arm ACE-I^c^	50	0.55 [0.36, 0.99]	0.20 [0.09, 0.56]	0.65 [0.42, 0.99]	0.045	0.63 [0.41, 0.96]	0.032	0.68 [0.45, 1.02]	0.065
Intensive BP arm CCB^d^	24	0.43 [0.29, 1.19]	0.49 [0.19, 1.25]	1.49 [0.88, 2.51]	0.14	1.43 [0.85, 2.41]	0.18	1.6 [0.96, 2.65]	0.069
Intensive BP arm Beta-Blocker^e^	51	0.69 [0.44, 1.48]	0.38 [0.13, 0.80]	0.77 [0.51, 1.17]	0.22	0.77 [0.50, 1.17]	0.22	0.84 [0.56, 1.26]	0.40

*ACE-I* angiotensin-converting enzyme inhibitor, *CCB* calcium channel blocker, *IQR* interquartile range.

^a^= +adjusted for baseline PCR.

^b^= +adjusted for baseline age, sex, estimated glomerular filtration rate, and systolic (BP).

^c^Ramipril.

^d^Amlodipine.

^e^Metoprolol.

Intensive BP lowering was overall more effective in lowering proteinuria compared to usual BP control, regardless of the BP agent (Table 1). Intensive BP control and beta-blockade were associated with a non-statistically significant 23% reduction in proteinuria compared to the reference group. Furthermore, intensive BP control + ACE-I use had a 35% greater reduction in proteinuria over 6 months compared to the reference group (Table 1). This effect of intensive BP control + ACE-I remained statistically significant compared to the reference group in adjusted Model 1 analyses. Use of a combination of intensive BP control and CCB was associated with a larger proportional increase in proteinuria (Table 1), though this effect was attenuated compared to what was observed in the usual BP control and CCB group.

Overall, our results suggest that ACE-I and beta-blockers achieved similar degrees of suppression of proteinuria when patients were already receiving an intensive blood pressure lowering regimen. However, there was an additive and synergistic effect of adding intensive BP control to ACE-I or beta-blockers compared to usual BP control. Furthermore, use of a CCB as previously known in the literature, was associated with a greater increase in proteinuria.

The strength of our study was our ability to quantify the additive effects of intensive BP control on ACE-I and other anti-hypertensive agents within a randomized setting. There are a few studies to our knowledge which have tested such BP interventions in a factorial design trial. There are a few limitations to our study. Our findings may not apply to patients in the general population who may differ from trial participants. Second, the AASK Trial only recruited Black participants, and findings may not apply to those of other racial or ethnic groups. Third, we do not have data on whether the participants had hyperaldosteronism and its role on treatment effects or suppression of proteinuria. Fourth, only a subset of patients enrolled in the AASK Trial had substantial proteinuria. However, studies have shown that even small degrees of change in proteinuria are associated with a higher risk of adverse outcomes [10]. Our data suggest the potential benefit of applying both intensive BP control with ACE-I or beta-blockers to suppress proteinuria in patients with chronic kidney disease.
